# Recurrent penile ulcer in an HIV-positive young man: An atypical presentation of secondary syphilis

**DOI:** 10.1016/j.jdcr.2026.02.012

**Published:** 2026-02-13

**Authors:** NagaNitya Vangala, Adarshlata Singh, Bhushan Madke, Varun H

**Affiliations:** Department of Dermatology, Venereology & Leprosy, Jawaharlal Nehru Medical College, Datta Meghe Institute of Higher Education & Research, (Deemed to be University), Wardha, Maharashtra, India

**Keywords:** generalized lymphadenopathy, genital ulcer disease, HIV coinfection, recurrent penile ulcer, secondary syphilis, skin of color

## Case

**Fig 1:**
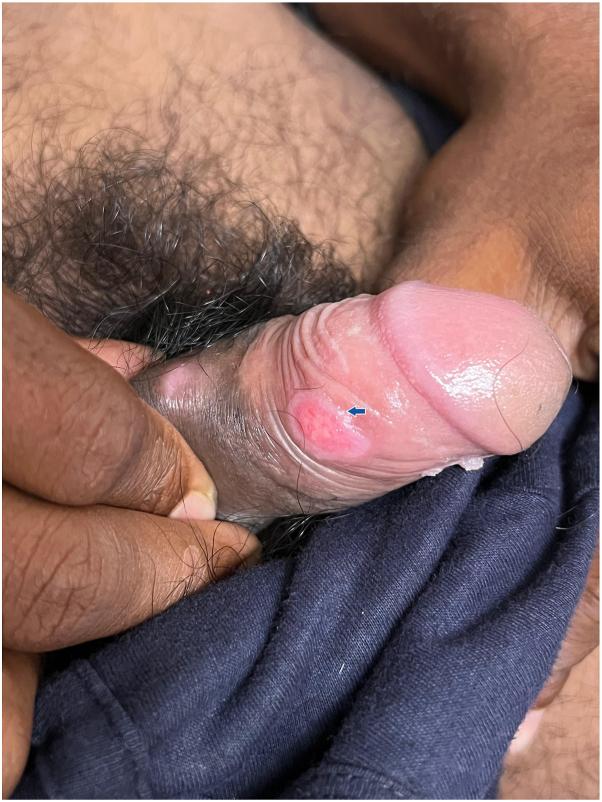
A well-defined, painless ulcer over the shaft of the penis with a clean base and indurated margins (*blue arrow*) in a young HIV-positive man, accompanied by generalized nontender lymphadenopathy, consistent with an atypical presentation of secondary syphilis. A wart was also noted on the ventral aspect of the penis.

**Fig 2:**
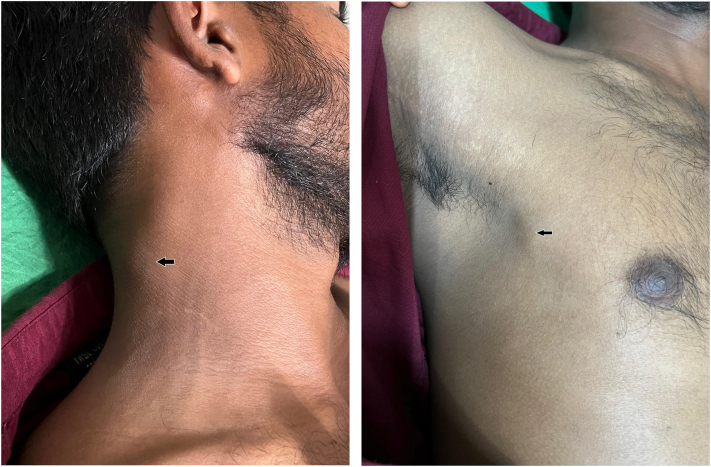
Enlarged, discrete, nontender cervical and axillary lymph nodes (*black arrow*) in the same patient, highlighting the generalized lymphadenopathy characteristic of secondary syphilis in the setting of HIV infection.

A man in his early 20s presented with a 3-week history of a painless ulcer over the shaft of the penis, with a history of recurrent similar episodes in the past. He reported multiple female sexual partners, his most recent sexual contact occurring 1 month prior to presentation. He also complained of swelling in the inguinal, cervical, and axillary regions, consistent with generalized lymphadenopathy. Serological testing revealed HIV seropositivity. Cutaneous examination showed a well-defined ulcer with a clean base and indurated margins over the penile shaft (Fig 1), along with multiple firm, nontender lymph nodes in the inguinal, cervical, and axillary regions (Fig 2). The patient reported 2 prior episodes of similar painless penile ulcers over the preceding year, occurring at different sites along the penile shaft, including a more proximal site, which resolved spontaneously without scarring or any treatment.

## Question: A young HIV-positive man with high-risk sexual behavior presents with a recurrent, painless ulcer over the penile shaft and generalized lymphadenopathy. Which of the following findings would most strongly support the diagnosis suggested by the clinical image?

A. Painful grouped vesicles with multinucleated giant cells on Tzanck smear
B. A high-titer non-treponemal serologic test with positive treponemal confirmation
C. Donovan bodies on Wright–Giemsa stain
D. Stellate necrotic ulcers with purulent base and tender buboes
E. Sharply demarcated violaceous plaque recurring at the same site after drug intake

Correct answer: B. A high-titer non-treponemal serologic test with positive treponemal confirmation.

## Answer discussion

The clinical presentation of a recurrent, painless penile ulcer accompanied by generalized lymphadenopathy in a young man with HIV infection and high-risk sexual behaviour is most consistent with early infectious syphilis with overlapping primary and secondary stages. Although a solitary, self-healing chancre is the classic manifestation of primary syphilis, individuals living with HIV frequently demonstrate atypical and exaggerated clinical patterns, including multiple, persistent, or recurrent genital ulcers and early dissemination.^1^, ^2^, ^3^

Secondary syphilis results from hematogenous spread of *Treponema pallidum* and is characterized by generalized, nontender lymphadenopathy, mucocutaneous lesions, and systemic involvement.^1^^,^^3^ Genital ulceration may persist or recur in this stage, particularly in immunocompromised hosts, leading to diagnostic confusion with other causes of genital ulcer disease such as herpes simplex infection, chancroid, lymphogranuloma venereum, and fixed drug eruption.^1^^,^^4^

Among the options provided, a high-titer non-treponemal serologic test (Venereal Disease Research Laboratory or Rapid Plasma Reagin) with confirmatory treponemal positivity (Treponema pallidum Hemagglutination Assay or Fluorescent Treponemal Antibody-Absorption) most strongly supports the diagnosis.^1^^,^^2^^,^^4^ Non-treponemal titers typically peak during secondary syphilis and correlate with disease activity, while treponemal tests establish exposure to *T. pallidum*.^1^^,^^3^ In contrast, painful grouped vesicles with multinucleated giant cells suggest herpes simplex virus infection; Donovan bodies are diagnostic of granuloma inguinale; stellate necrotic ulcers with tender buboes are characteristic of chancroid; and a sharply demarcated recurrent violaceous plaque favours a fixed drug eruption.^2^^,^^4^

Recognition of such atypical presentations is crucial, as syphilis and HIV frequently coexist and potentiate each other’s transmission.^1^^,^^3^ Early diagnosis using serologic testing, supported by clinical pattern recognition, allows prompt treatment and prevents progression to late complications and further spread.^1^^,^^4^

## Teaching points

• Syphilis in people living with HIV may present atypically, with recurrent or persistent genital ulcers and overlapping clinical stages.^1^^,^^3^
• Generalized, nontender lymphadenopathy is a key bedside clue pointing toward secondary syphilis in the setting of genital ulcer disease.^1^
• A high-titer non-treponemal test with treponemal confirmation remains the most reliable investigation to support the diagnosis in such cases.^1^^,^^4^

## Conflicts of interest

None disclosed.
